# Far upstream element-binding protein 1 is a prognostic biomarker and promotes nasopharyngeal carcinoma progression

**DOI:** 10.1038/cddis.2015.258

**Published:** 2015-10-15

**Authors:** Z-H Liu, J-L Hu, J-Z Liang, A-J Zhou, M-Z Li, S-M Yan, X Zhang, S Gao, L Chen, Q Zhong, M-S Zeng

**Affiliations:** 1 Sun Yat-sen University Cancer Center, State Key Laboratory of Oncology in South China, Collaborative Innovation Center for Cancer Medicine, Guangzhou, China; 2 Department of Experimental Research, Sun Yat-sen University Cancer Center, Guangzhou, China; 3 Collaborative Innovation Center of Cancer Medicine, National Institute of Biological Sciences, Beijing, China; 4 Department of Biotherapy, Sun Yat-sen University Cancer Center, Guangzhou, China; 5 National Institute of Biological Sciences, Beijing, China

## Abstract

Nasopharyngeal carcinoma (NPC) is a malignant epithelial tumor with tremendous invasion and metastasis capacities, and it has a high incidence in southeast Asia and southern China. Previous studies identified that far upstream element-binding protein 1 (FBP1), a transcriptional regulator of c-Myc that is one of the most frequently aberrantly expressed oncogenes in various human cancers, including NPC, is an important biomarker for many cancers. Our study aimed to investigate the expression and function of FBP1 in human NPC. Quantitative real-time RT-PCR (qRT-PCR), western blot and immunohistochemical staining (IHC) were performed in NPC cells and biopsies. Furthermore, the effect of FBP1 knockdown on cell proliferation, colony formation, side population tests and tumorigenesis in nude mice were measured by MTT, clonogenicity analysis, flow cytometry and a xenograft model, respectively. The results showed that the mRNA and protein levels of FBP1, which are positively correlated with c-Myc expression, were substantially higher in NPC than that in nasopharyngeal epithelial cells. IHC revealed that the patients with high FBP1 expression had a significantly poorer prognosis compared with the patients with low expression (*P*=0.020). In univariate analysis, high FBP1 and c-Myc expression predicted poorer overall survival (OS) and poorer progression-free survival. Multivariate analysis indicated that high FBP1 and c-Myc expression were independent prognostic markers. Knockdown of FBP1 reduced cell proliferation, clonogenicity and the ratio of side populations, as well as tumorigenesis in nude mice. These data indicate that FBP1 expression, which is closely correlated with c-Myc expression, is an independent prognostic factor and promotes NPC progression. Our results suggest that FBP1 can not only serve as a useful prognostic biomarker for NPC but also as a potential therapeutic target for NPC patients.

Nasopharyngeal carcinoma (NPC) presents a remarkable geographic pattern and has a high incidence in Southern China, Hong Kong, Taiwan and Southeast Asia.^1, 2^ NPC is associated with Epstein–Barr virus (EBV) infection.^3, 4, 5, 6^ Serum EBV antibodies and EBV-DNA serves as a useful tools for the diagnosis in NPC.^7, 8, 9^ Although there have been improvements in diagnosis techniques, irradiation and chemo-radiotherapy, local recurrence or distant metastasis are the main reasons for treatment failure.^10^ Therefore, it is particularly important to look for diagnostic markers and therapeutic targets for NPC. c-Myc is one of the most frequently dysregulated oncogenes in various human cancers, including NPC^11^ and its expression is induced by EBV-encoded genes,^12^ such as LMP1 (ref. 13) and LMP2A.^12, 14, 15^ The aberration of c-Myc in cancers could occur at different levels, such as genetic amplification, upregulation of transcription, post-transcriptional regulation and post-translational regulation.^16, 17, 18, 19^ It has been shown that gain of chromosome 8, where c-Myc is localized, was observed on 30% of primary NPCs. However, overexpression of the c-Myc mRNA or protein has been observed in ~80–90% of NPC samples.^20^ Thus, transcriptional or post-transcriptional regulation of c-myc may be the major mechanism for its aberrant expression in NPC. The far upstream element (FUSE)-binding proteins (FBPs) are a family of three regulatory proteins, termed FBP1, FBP2 and FBP3.^21^ FBP1 was first identified as a DNA-binding protein that regulates the expression of c-Myc by binding to a single-stranded FUSE located upstream of the c-Myc promoter^22, 23^ FBP1 is also an RNA-binding protein that binds to the Hepatitis C and Enterovirus 71 RNAs and mediates their replication during retroviral infection.^24, 25^ FBP1 modulates viral replication by interacting with the untranslated regions of the Japanese encephalitis virus RNA.^26^ In apoptotic cells, FBP1 may translocate from the nucleus to the cytoplasm. This location is associated with the fact that FBP1 may be cleaved by caspase-3 and -7.^27^ Recently, FBP1 has been indentified as a potential biomarker for oligodendrogliomas,^28^ non-small cell lung cancer,^29^ breast cancer,^30, 31^ liver cancer,^32, 33^ colon cancer^34, 35^ and clear cell renal cancer.^36^ However, little is known about the prognostic significance and function of FBP1 and its role in the regulation of c-Myc in NPC.

In this study, we investigated FBP1 expression in NPC tissues using immunohistochemistry (IHC) and analyzed the correlation between FBP1, c-Myc and the clinicopathological characteristics. In addition, we also explored the function of the FBP1 protein in NPC cells using two independent small interference RNAs (siRNAs) to knock down the expression of FBP1, and found that silencing FBP1 suppresses cell proliferation, colony-formation abilities, side populations (SPs) and markedly reduces tumorigenesis in nude mice. Taken together, these results indicate that FBP1 was overexpressed in NPC and had an important role in the development of NPC.

## Results

### Expression of FBP1 positively correlates with c-Myc in NPC cells and tissues

**Figure 1:**
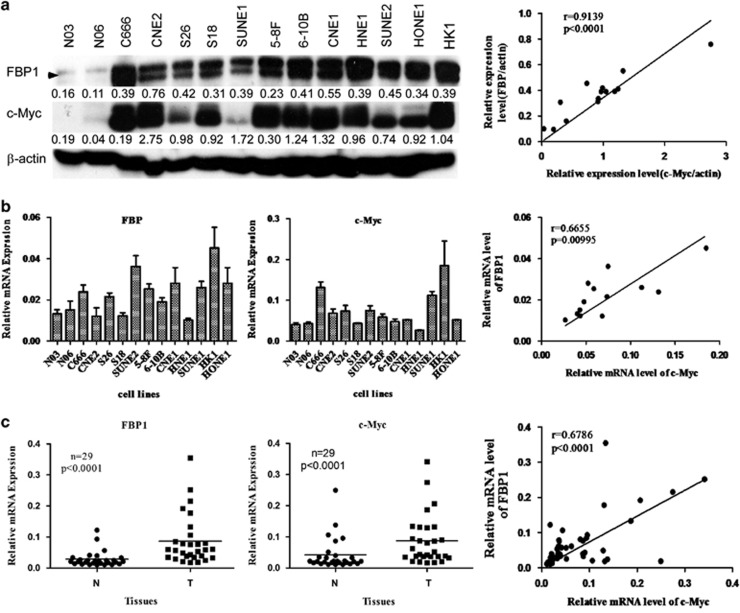
The expression of FBP1 directly correlates with c-Myc in nasopharyngeal carcinoma. (**a**) Western blots of normal human nasopharyngeal epithelial cell samples (N03 and N06) and samples of nasopharyngeal carcinoma cell lines using antibodies against FBP1 and c-Myc (left panel). The relationship between FBP1 and c-Myc is shown in the right panel. *β*-Actin was used as a control for protein loading and integrity. (**b**) FBP1 and c-Myc mRNA levels in a series of NPC cell lines compared with the normal human nasopharyngeal epithelial cells. mRNA levels are presented as the means±S.D. and are normalized to the housekeeping gene *β*-actin using qRT-PCR. (**c**) The relative expression of FBP1 and c-Myc in 29 pairs of matched NPC and non-tumor tissues at the transcript level (left and middle panels). The right panel shows the association between FBP1 and c-Myc

To explore the expression of FBP1 in NPC cells and tissues, we first analyzed the levels of the FBP1 protein and mRNA in two non-cancerous primary human nasopharyngeal epithelial cells and 12 NPC cell lines by western blot and quantitative real-time PCR (qRT-PCR). As shown in Figures 1a and b, the levels of the FBP1 protein and transcript were much higher in NPC cells than in normal cells. It has been reported that FBP1 is the major transcriptional regulator for c-Myc, and there is a correlation between FBP1 and c-Myc expression in many cancers.^37^ Therefore, we also analyzed c-Myc expression in these samples. As expected, the expression of c-Myc was consistent with FBP1. Furthermore, we used the Spearman's rank correlation analysis to examine the relationship between FBP1 and c-Myc expression and found that FBP1 expression was positively associated with c-Myc at the protein (Spearman's *r*=0.9137, *P*<0.0001; Figure 1a, right panel) and mRNA levels (Spearman's *r*=0.6655, *P*=0.00095; Figure 1b, right panel).

To further determine the expression of FBP1 and c-Myc in NPC tissue samples, we analyzed the transcript levels of FBP1 and c-Myc in 29 NPC tissues and 29 adjacent non-cancerous tissues by qRT-PCR. We observed that the expression level of FBP1 and c-Myc in NPC tissues was significantly higher than in non-cancerous tissues (Figure 1c), and also found that the expression of FBP1 correlated with c-Myc (Spearman's *r*=0.6786, *P*<0.0001; Figure 1c, right panel). Taken together, these data demonstrated that FBP1 was frequently upregulated and its expression was positively associated with c-Myc in both NPC cells and tissues.

### Inhibition of FBP1 expression suppresses NPC cell proliferation and clonogenicity

**Figure 2:**
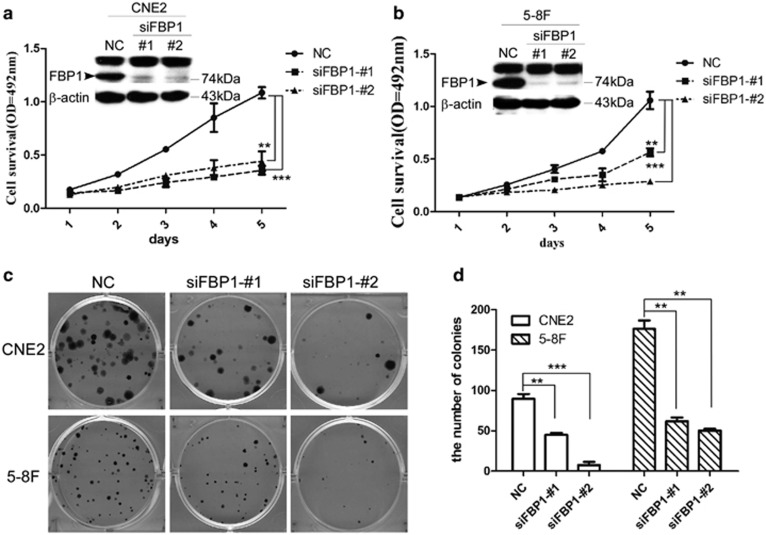
FBP1 silencing suppressed the proliferation and clone formation of NPC cells. (**a, b**) Western blot samples from CNE2 (**a**) and 5-8F (**b**) cells were transfected with NC- or FBP-targeting (KD) siRNAs. *β*-Actin was used as an internal control (upper panel). The MTT assay measured the viability of CNE2 and 5-8F cells with NC- or FBP1-targeting (KD) siRNAs. The data represent the means±S.E.M. (lower panel; **P<0.01, ***P<0.001 by the paired *t*-test). (**c, d**) The representative pictures (**c**) and the quantitative analysis (**d**) of colony-formation assay of CNE2 and 5-8F cells transfected with NC- or FBP1-targeting (KD) siRNAs. The assay was performed in triplicate. The data represent the means± S.E.M. (**P<0.01, ***P<0.001 by the paired *t-*test)

To explore the role of the FBP1 gene in NPC, we used two independent siRNAs to knock down FBP1 expression in CNE2 and 5-8F cells. A significant reduction was observed at the transcript level (data not shown) and protein level (Figures 2a and b). We next performed the MTT assay to test whether knocking down the expression of FBP1 suppressed cell proliferation. The results showed that FBP1 knockdown reduced the tumor cell viability (Figures 2a and b, CNE2-NC *versus* siFBP1-1#, *P*<0.01; CNE2-NC *versus* siFBP1-2#, *P*<0.0001; 5-8F-NC *versus* siFBP1-1#, p<0.01; 5-8F-NC *versus* siFBP1-2#, P<0.001). In addition, we also observed that inhibition of FBP1 in CNE2 or 5-8F cells resulted in significantly fewer and smaller colonies compared with the control (Figures 2c and d, CNE2-NC *versus* siFBP1-1#, *P*<0.01; CNE2-NC *versus* siFBP1-2#, *P*<0.001; 5-8F NC *versus* siFBP1-1# and siFBP1-2#, *P*<0.01). All of these results suggested that the FBP1 protein could exhibit oncogenic functions in NPC.

### Knockdown of FBP1 inhibits the side population in NPC cell

**Figure 3:**
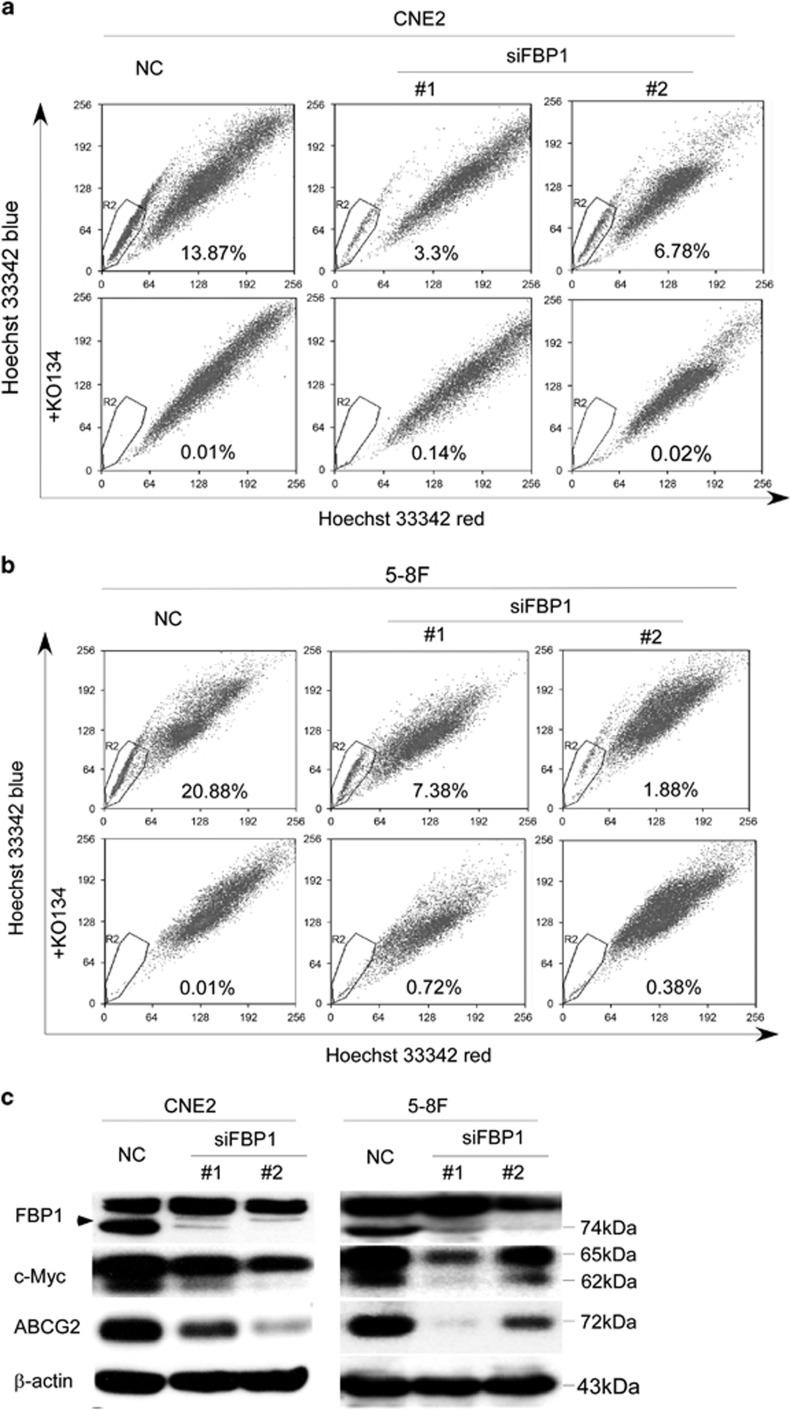
Knockdown of FBP1 decreased the side population in NPC cells. (**a**) Flow cytometry analysis of Hoechst 33342 staining was performed in CNE2 cells transfected with NC- or FBP1-targeting (KD) siRNAs (upper panel) and treated with ko143 (final concentration: 5 *μ*m) before Hoechst 33342 staining (lower panel). The percentage of SP cells is indicated. (**b**) Flow cytometry analysis of Hoechst 33342 staining was performed and 5-8F cells transfected with NC- or FBP1-targeting (KD) siRNAs (upper panel) and treated with ko143 (final concentration: 5 *μ*m) before Hoechst 33342 staining (lower panel). The percentage of SP cells is indicated. (**c**) Representative western blots of FBP1, c-Myc, ABCG2 and *β*-actin in CNE2 and 5-8F cells transfected with NC- or FBP1-targeting (KD) siRNAs. *β-*Actin served as a loading control

It has been known that FBP1 is involved in carcinogenesis via c-Myc-independent or -dependent pathways.^32, 36, 38^ Several studies demonstrated that the ABC family is a direct transcriptional target of the Myc family.^39, 40, 41^ To further explore whether the FBP/MYC/ABCG2 pathway contributed to the induction of SP cells in NPC, we performed Hoechst 33342 flow cytometry and found an apparent decrease of the number of SP when FBP1 was knocked down (Figures 3a and b), whereas Ko143 (a specific inhibitor of ABCG2) obviously inhibited the efflux of Hoechst 33342. Moreover, an obvious reduction of the c-Myc and ABCG2 proteins was present in FBP silenced cells (Figure 3c). These results indicated that FBP1 may activate the c-Myc/ABCG2 pathway and induce an SP phenotype in NPC cells.

### FBP1 silencing reduces the chemosensitvity and radiosensitvity of NPC cells

**Figure 4:**
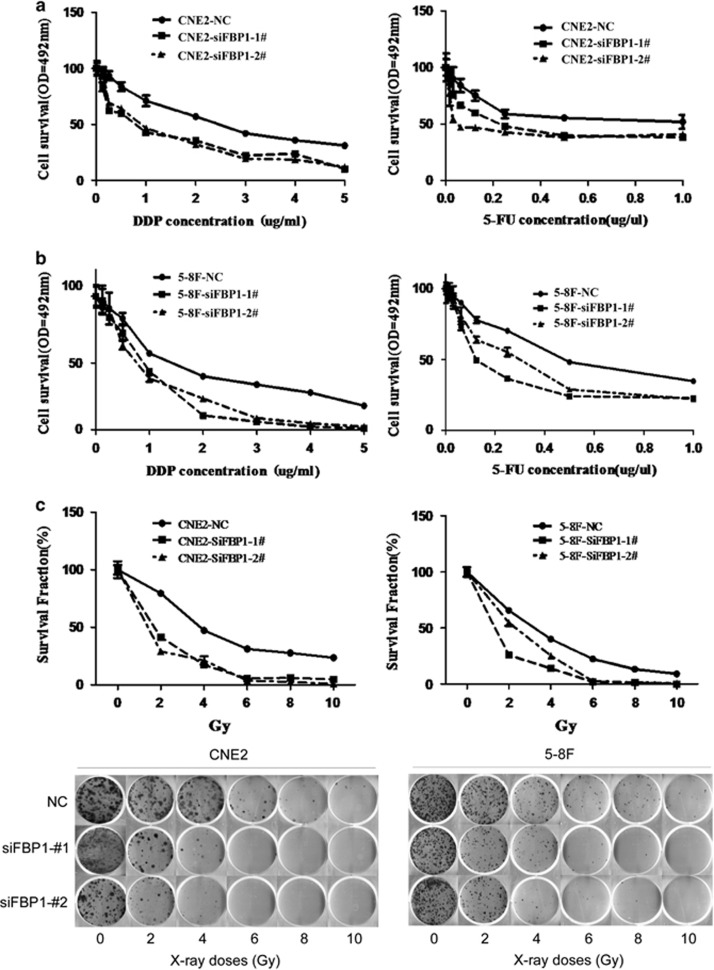
Results of radiation and chemotherapy sensitivity. (**a**) The growth curves of CNE2 cells transfected with NC- or FBP1-targeting (KD) siRNAs at 48 h post treatment with the indicated doses of DDP (left panel) and 5-FU (right panel). (CNE2-NC *versus* CNE2-siFBP1-1#1 and siFBP1-2#, *P*<0.001 by the paired *t*-test). (**b**) The growth curves of 5-8F cells transfected with NC- or FBP1-targeting (KD) siRNAs at 48 h post treatment with the indicated doses of DDP (left panel) and 5-FU (right panel). (5-8F-NC *versus* 5-8F-siFBP1-1# and 5-8F-siFBP1-2#, *P*<0.05, by the paired *t-*test). (**c**) The survival fraction (SF) curves (upper panel) and the representative pictures of CNE2 and 5-8F cells transfected with NC- or FBP1-targeting (KD) siRNAs at 10 days after X-ray irradiation with the indicated Gy doses (lower panel). (NC *versus* siFBP1-1#1 in CNE2 and 5-8F, *P*<0.05; NC *versus* siFBP1-2# in CNE2 and 5-8F, *P*<0.01 by the paired *t*-test)

The SP cells that exhibited cancer stem cell characteristics have been reported in NPC.^42, 43^ As we showed that inhibition of FBP1 expression could reduce the ratio of CSC-like SP, we explored whether FBP1 also had a role in tumor resistance to chemotherapy and radiotherapy. Two common chemotherapeutic drugs (cisplatin (DDP); 5-flurouracil (5-FU)) for NPC were used to examine the cellular response after suppressing FBP1 expression. The results showed that suppressing FBP1 expression increased the tumors' sensitivity to both DDP and 5-FU (Figures 4a and b). Radiotherapy is a main treatment for NPC. Thus, we treated the cells that were transfected with the siRNA duplexes with different doses and observed that knocking down of FBP1 decreased the radioresistance in NPC cells. (Figure 4c; CNE2-NC *versus* CNE2-siFBP1-1#1, *P*<0.05; CNE2-NC *versus* CNE2-siFBP1-2#, *P*<0.01; 5-8F-NC *versus* 5-8F-siFBP1-1#, *P*<0.0264; 5-8F-NC *versus* 5-8F-siFBP1-2#, *P*<0.01). Together, targeting FBP1 can reduce both chemotherapy and radiotherapy resistance.

### Immunohistochemical analysis of FBP1 or c-Myc expression and their correlations with clinicopathological characteristics

**Figure 5:**
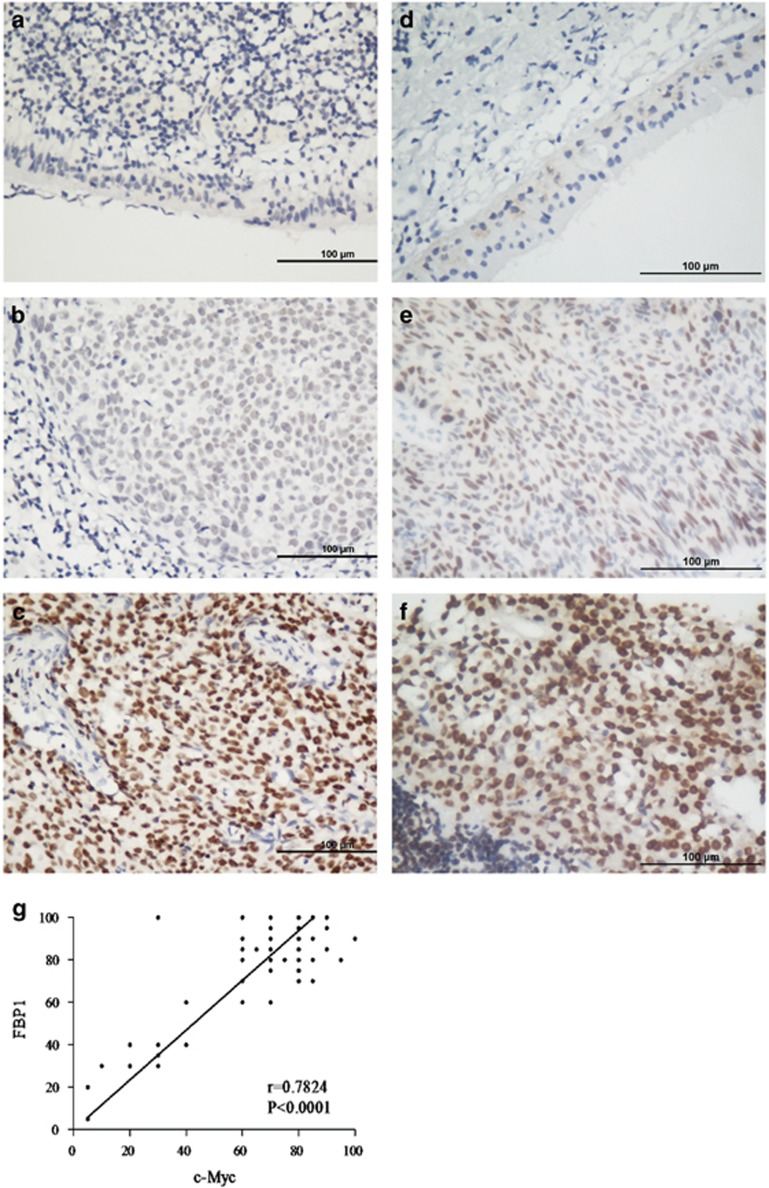
The expression of FBP1 in nasopharyngeal carcinoma (NPC) by immunohistochemical (IHC) staining Positive expression of FBP1 was primarily detected in the nucleus of the NPC cells. (**a**) Negative expression of FBP1 in normal nasopharyngeal tissues. (SP x400, left upper panel). (**b**) Moderate expression of FBP1 in NPC. (SP x400, right upper panel). (**c**) High expression of FBP1 in NPC. (SP x400, right lower panel). (**d**) Negative expression of c-Myc in normal nasopharyngeal tissues. (SP x400, left upper panel). (**e**) Moderate expression of c-Myc in NPC. (SP x400, right upper panel). (**f**) High expression of c-Myc in NPC. (SP x400, right lower panel). (**g**) The correlation of FBP1 and c-Myc expression by IHC

To reveal a potential role for FBP1 in NPC, 83 NPC samples from patients were stained with the human FBP1 antibody and c-Myc antibody using IHC. We found that both FBP1 and c-Myc were predominantly located in the nuclei of tumor cells in the NPC samples (Figure 5). Representative images of FBP1 and c-Myc IHC staining in NPC tissues are shown in Figures 5a–f. The normal nasopharyngeal epithelial cells has no or very low staining intensity of FBP1 (Figure 5a) and c-Myc (Figure 5d). The non-stained area is constituted by stromal cells and lymphocyte, suggesting that the IHC staining of FBP1 (Figure 5b and c) and c-Myc (Figure 5e and f) are specific to NPC cells. In addition, we also observed that FBP1 expression is positively associated with c-Myc^44^ (*r*=0.7824, *P*<0.001, Figure 5g). We further analyzed the correlation between the FBP1 and c-Myc expression and clinicopathological characteristics. As presented in Table 1, both FBP1 and c-Myc expression correlated with the clinical stage (*P*=0.010). In addition, c-Myc was also associated with N classification (*P*=0.040), whereas FBP1 was associated with locoregional failure (*P*=0.049) and distant metastasis (*P*=0.038). However, there were no significant correlations with other clinical features, including age, gender, T classification, EBV-DNA, EBV-VCA-IgA, EBV-EA-IgA, tumor progression and death.

### Relationship between FBP1 or c-Myc and patient survival

**Figure 6:**
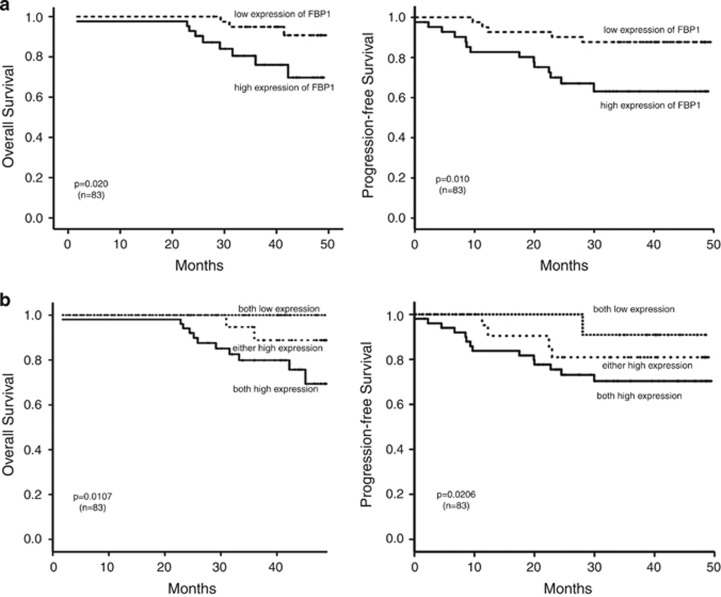
Relationship between FBP1 and patient survival. (**a**) Overall survival curves and progression-free survival curves of patients with low and high FBP1 expression. (**b**) Overall survival curves and progression-free survival curves of patients with high expression of both FBP1/c-Myc, low expression of both FBP1/c-Myc and high expression of either FBP1 or c-Myc

We carried out the Kaplan–Meier test to analyze the association between the expression of FBP1 and patient survival. Patients with low FBP1 expression have longer OS (*P*=0.020; Figure 6a, left panel). Consistently, progression-free survival (PFS) was shorter in the FBP1 high expression group than in those with low FBP1 expression (*P*=0.010, Figure 6a, right panel). We further analyzed the combined the phenotypes of FBP1 and c-Myc and found that patients with low expression of both FBP1 and c-Myc have longer OS and PFS than those with high levels of expression of both FBP1 and c-Myc (Figure 6b). The clinicopathological variables that were relevant to the OS are presented in Supplementary Figure S1.

Next, we performed univariate and multivariate analyses using the COX proportional hazards model to investigate whether the gender, age, T stage, N stage, clinical stage, FBP1 or c-Myc expression were correlated with OS and PFS. As shown in Table 2, FBP1 was a prognostic factor for OS and PFS. The T stage and clinical stage were prognostic factors for OS, whereas gender was a prognostic factor for PFS. From the multivariate analyses, we found that both the clinical stage and FBP1 expression were independent indicators for both OS and PFS (Table 3).

### Repression of FBP1 inhibits tumorigenesis in nude mice

**Figure 7:**
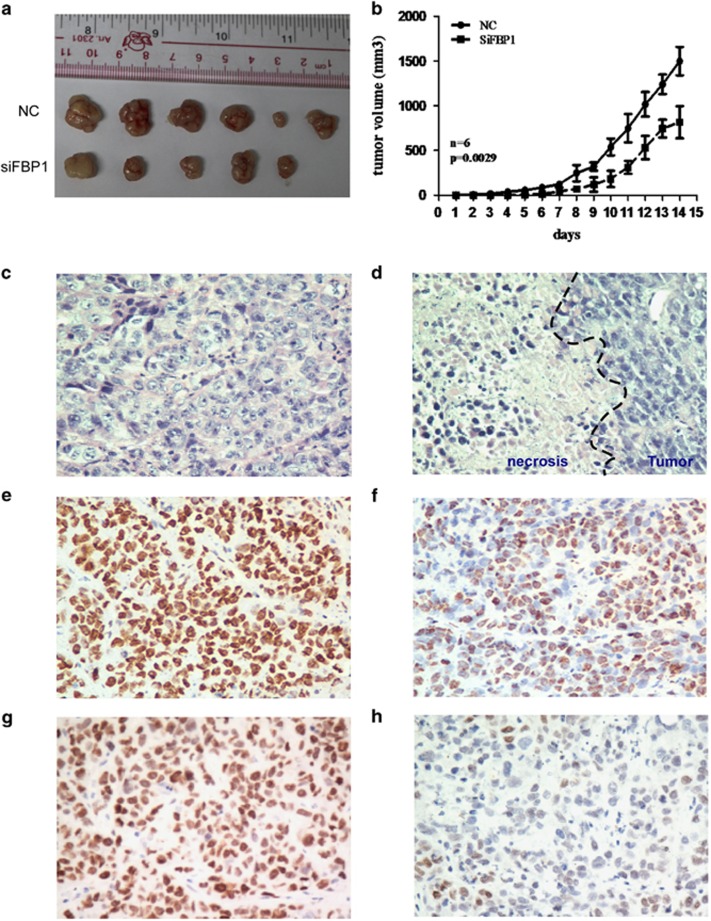
FBP1 silencing reduced the tumorigenicity of NPC cells. (**a**) Images of the tumors formed by CNE2 cells transfected with NC or siFBP1. (**b**) The growth curve of tumors formed by CNE2 cells transfected with NC or siFBP1. The data are presented as the means±S.D. (*n*=6 mice in each group). (**c**–**d**) HE staining pictures of mice treated with NC (**c**) or with siFBP1 (**d**) (SP x400). (**e**–**f**) IHC staining pictures of FBP1 expression in the primary xenografts from cells treated with NC (**e**) or with siFBP1 (**f**) (SP x400). (**g**–**h**) IHC staining pictures of c-Myc expression in the primary xenografts from cells treated with NC (**g**) or with siFBP1 (**h**) (SP x400)

To further analyze the role of FBP1 in the progress of NPC, CNE2 cells, which were treated with the siRNA targeting FBP1, were injected into the subcutaneous area of 5-week-old female BALB/c nude mice. Tumor formation was assessed after 2 weeks. The repression of FBP1 reduced the volume and weight of the xenografts (Figures 7a and b, NC *versus* siFBP1, *P*=0.0029) The tumors obtained from injection of CNE2-NC/SiFBP1-1# were performed to assess the tumor pathology by HE staining (Figures 7c and d), FBP1 IHC staining (Figures 7e and f) and c-Myc IHC staining (Figure 7g and h), and then analyzed by a pathologist. We found that there were lymphocyte infiltrate and necrosis in the xenografts of siFBP group (Figure 7d), whereas the intensity of FBP1 and c-Myc was weak in the xenografts of siFBP group compared with that in NC group (Figures 7f and h). In summary, our data suggested that knockdown of FBP1 repressed the tumor progression in nude mice, probably attributable to the downregulation of c-Myc expression.

## Discussion

In the present study, it was the first time to show that the expression of FBP1 was higher in NPC cell lines and NPC tissues than that in primary NPECs and normal nasopharyngeal tissues (Figure 1). In addition, the patients with high levels of FBP1 have a lower OS time than patients with low FBP1 expression, and there is a strong correlation between FBP and c-Myc expression.

c-Myc is located at 8q24.21, and encodes a transcription factor that has essential roles in cell proliferation, cell growth, differentiation and apoptosis.^45^ It has been reported that c-Myc is an oncogene and transcription factor involved in the tumorigenesis of multiple cancers.^46, 47, 48, 49, 50, 51^ Fan, CS *et al*^52^ indicated that c-Myc overexpression is a frequent genetic abnormality in NPC. Several studies showed that c-Myc induces the proliferation, migration and invasion of NPC cells.^53, 54, 55^ In addition, c-Myc was reported to promote radioresistance in a stem cell-like population of NPC cells.^19^ Mounting evidence indicates that c-Myc expression in the tumor may be an important strategy for designing appropriate treatment, using lower drug dosages and overcoming drug resistance and drug-related toxicity.^56, 57, 58, 59^ Hence, many investigators and international pharmaceutical companies have turned to identifying c-Myc inhibitors to cure cancers. However, the development of c-Myc inhibitors for clinical applications has not been very successful to date. Thus, a potential strategy may be to target the major protein that regulates c-Myc expression rather than only targeting c-Myc itself.

The FUSE-binding protein 1 (FBP1) was first discovered as transcriptional regulator of the proto-oncogene c-Myc. FBP1 regulated the expression of c-Myc by binding to the FUSE in the c-Myc promoter region at 1.5-kb upstream of the transcription start site.^22^ A number of reports demonstrate that FBP1 is involved in the regulation of cellular processes, including gene expression, differentiation and apoptosis.^22, 34, 38, 60, 61^ Many studies suggest that FBP1 is a proto-oncogene, because there is a close relationship between FBP1 expression and tumor development. In our study, we showed that the expression of FBP1 is positively correlated to the progress of NPC. Moreover, we also found that patients with high levels of the FBP1 protein had poorer OS and PFS (Figure 6a). In addition, higher expression of both the FBP1 protein and the c-Myc protein were remarkably correlated with patients' shorter survival time (Figure 6b). This result is consistent with a previous study in renal carcinomas.^36^

Previous study showed that FBP1 binds to c-Myc promoter and regulates its expression and effects its downstream targets.^22, 23, 62^ Downregulation of c-Myc reduced the cyclin-dependent kinase (Cdk) 4/6 activity and proliferating cell nuclear antigen (PCNA), Cdc2 and Rb-binding protein,^63^ causing cell cycle arrest at the G1 phase.^64^ In this study, we explored the function of FBP1 in cell proliferation and clonality using siRNA in CNE2 and 5-8F cells, and found that knockdown of FBP1 decreased the expression of c-Myc, then significantly inhibited the cell proliferation and clone formation by MTT assay and clonogenicity analysis, suggesting that FBP1 promotes NPC cell proliferation and colony formation ability, probably through c-Myc (Figure 2).

Several studies demonstrated that Myc can directly regulate the ATP-binding cassette drug transporters, which shuttle hydrophobic lipophilic compounds across the membranes in an ATP-dependent manner.^40, 65, 66^ However, there has been no evidence showing that FBP1 induces tumorigenesis through the enhancement of cancer stem-like cell (CSC). Here we first reported that the ratio of the SP in NPC was reduced after knocking down FBP1 expression by siRNA (Figures 3a and b). Western blot analysis suggested that FBP1 may induce the CSC-like characteristics by activating the FBP/C-Myc/ABCG2 pathway. It is known that CSC contributes to the resistance to irradiation and chemotherapy.^43^ Knockdown of FBP1 consistently reduced the chemoresistant and radioresistant characteristics of NPC cells (Figure 4).

In summary, our study demonstrated that FBP1 is a novel biomarker and can serve as a potential predictor for tumor progression in NPC. In addition, FBP is strongly correlated with c-Myc expression, and knocking down FBP1 expression could suppress NPC progression *in vitro* and *in vivo*, which suggested that FBP1 could be a potential therapeutic target in reducing chemoresistance and radioresistance as well as reducing tumor progression in NPC.

## Materials and Methods

### Patients and tissue specimens

Twenty-nine NPC biopsy samples and an equal number of non-cancerous nasopharyngeal tissues were acquired from Sun Yat-sen University Cancer Center (SYSUCC), Guangzhou, China and used for qRT-PCR. For IHC analysis, the paraffin-embedded NPC specimens were acquired from 83 patients (median age: 46 years,range 24–77 years) diagnosed with NPC in 2009–2013 at Cancer Center, Sun Yat-sen University, Guangzhou, China. This study was approved by the Institutional Research Ethics Committee at the Cancer Center. The clinical characteristics of the NPC patients were described in Table 1.

### Cell culture

Normal primary NPECs^67, 68, 69^(NPEC03 and NPEC06) were cultured in Keratinocyte serum-free medium (Invitrogen, Carlsbad, CA, USA). The NPC cell lines^70, 71^ (C666, CNE2, S18, S26, SUNE2, 5-8F, 6-10B, SUNE1, CNE1, HNE1, HK1 and HONE1) were cultured in RPMI 1640 (Invitrogen) supplemented with 10% fetal bovine serum (Hyclone, Logan, UT) in a humidified 5% CO_2_ incubator at 37 °C.

### siRNA transfection

The siRNAs targeting the mRNA of human FBP1 (Gene ID: 8880 NM_001303433.1) were denoted as siFBP1-#1 (GGUCAAGGCAACUGGAACA) and siFBP1-#2 (GGACAGGUUGAUUAUACCA). The negative control (NC)^72, 73, 74^ RNA duplex for the siRNA was indicated as NC and was not homologous to any human genome sequences. The CNE2 and 5-8F cells were plated in 6-well plates for 18 h, and then transfected with the 20 nM of the RNA duplex and 5 *μ*l Lipofectamine RNAiMAX (Invitrogen), according to the manufacturer's instructions. After 48 h, the cells were harvested for further experiments, including qRT-PCR, western blotting, MTT, colony formation assay and nude mice xenograft assay.

### RNA isolation and reverse transcriptase PCR analysis

Total RNA was extracted from the tissue specimens and NPC cell lines using the TRIzol reagent (Invitrogen), according to the manufacturer's instructions. The reverse transcriptase kit (Promega, Madison, Wisconsin, USA,) was used to synthesize the complementary DNA from 2 *μ*g of the total RNA. qRT-PCR was performed using the Power SYBR Green qPCR SuperMix-UDG (Invitrogen) to detect the mRNA level of the target genes using a LightCycler 480 II (Roche, Basel, Swiss). *β-*Actin was used as an internal control. The relative expression of TACC3 was normalized to the expression of *β*-actin, which yielded a 2-▵ct value. All reactions were performed in triplicate in three independent experiments. The sequences of the real-time PCR primers were as follows:

FBP1 sense: 5′-GGAGGTGATGCAGGGACATC-3′

FBP1 antisense: 5′-CTGCTGATGCATCGGTGGTA-3′

*β*-actin sense: 5′-CGCGAGAAGATGACCCAGAT-3′

*β*-actin antisense: 5′-GGGCATACCCCTCGTAGATG-3′.

### Western blotting analysis

Cells were collected and lysed in SDS sample buffer (62.5 mM Tris-HCl (pH 6.8), 3% SDS, 10% glycerol, 50 mM DL-dithiothreitol and 0.1% bromophenol blue) with protease inhibitors (Roche, Indianapolis, IN, USA). The protein concentrations were determined by the BCA method (Pierce, Thermo Fisher Scientific, Rockford, IL, USA). The proteins (20 *μ*g) were separated by SDS-PAGE and transferred to a polyvinylidene difluoride membrane. Bovine serum albumin (BSA; 5%) in TBS-T (1 mol/l Tris-HCl (pH 7.5), 0.8% NaCl and 0.1% Tween 20) was used to block the membrane. Then, the membrane was incubated with anti-FBP1 (Santa-Cruz, sc-374342, Dallas, TX, USA), anti-c-Myc (Cell Signaling, #9402, Danvers, MA, USA), anti-ABCG2 (Abcam, ab3380, Cambridge, MA, USA), and anti-*β*-actin (Sigma-Aldrich, A5441, St Louis, MO, USA) antibodies at 4 °C overnight. The blots were then treated with an HRP-conjugated secondary antibody (Pierce).

### 3-(4,5-Dimethylthiazol-2-yl)-2,5-diphenyltetrazolium bromide reduction (MTT) assay

The MTT assay was used to measure the viability of the NPC cells. Cells transfected with specific siRNA were seeded onto a 96-well plate at a density of 1000 cells per well. The cells were incubated with 0.2% MTT for 4 h at 37 °C, then 200 *μ*l dimethyl sulfoxide (DMSO) per well was added to the culture cells to dissolve the crystals, and the cells were counted daily by reading the absorbance at 490 nm. The concentration at which DDP or 5-FU produced 50% growth inhibition (IC50) was calculated by the relative survival curve. Cells transfected with specific siRNA were seeded onto a 96-well plate at a density of 1000 cells per well. After 8 h for adherence, cells treated with chemotherapy drugs such as DDP and 5-FU at the increasing concentrations for 48 h. Finally, about 4 h before test, MTT (5 mg/ml) was added into the medium and 200 *μ*l DMSO was added to dissolve the purple crystals. The 96-well plates were read at the wavelength of 490 nm.

### Colony formation assay

After transfection with the specific siRNAs, cells (200 cells per well) were seeded in a 6-well plate and cultured for 14 days in a humidified 5%CO_2_ incubator at 37 °C.

Colonies were fixed in methanol for 10 min, and then stained with 0.5% crystal violet for 15 min. All visible colonies were quantified. The radiation dose that produced 50% growth inhibition was calculated by the relative survival fraction. At least three independent experiments were performed for each assay.

### Detection of the side population of NPC cell lines

Forty-eight hours after transfection with specific siRNAs, CNE2 and 5-8F cells were trypsinized and resuspended at density of 1 × 10^6^ cells per ml. The DNA-binding dye, Hoechst 33342 (Sigma-Aldrich), was then added at a final concentration of 7.5 *μ*g/ml and incubated for 90 min in the dark with periodic mixing. After washing twice with PBS, the cells were placed at 4 °C in the dark before flow cytometry (EPICS ALTRA Flow Cytosorter, Beckman Coulter, Indianapolis, IN, USA) using dual-wavelength analysis. However, a fraction of the cell preparation was incubated with 5 *μ*M Ko143 (Ko143, a specific inhibitor of ABCG2, Sigma-Aldrich) for 10 min at 37 °C before adding Hoechst 33342 to determine whether Ko143 would block the fluorescent efflux of SP cell.

### Immunohistochemistry

The 4 *μ*m paraffin-embedded NPC sections were deparaffinized in xylene and an alcohol gradient to rehydrate the sections. Next, the sections were treated with a Citrate Antigen Retrieval Solution (pH=8.0) in a pressure cooker for 5 min. Subsequently, 5% BSA in PBS (25 mM Tris, 0.8% NaCl, 2.68 mM KCl (pH 7.4)) was added to block non-specific binding, and the sections were then incubated with a mouse monoclonal anti-FBP1 antibody (1 : 100, Santa-Cruz Biotechnology, sc-374342, Dallas, TX, USA) or anti-c-Myc antibody (1 : 200, Cell Signaling, #9402, Danvers, MA, USA) in a moist chamber overnight at 4 °C. The secondary antibodies were incubated for 30 min at 37 °C on the next day.

Finally, the sections were incubated in 3, 3-diaminobenzidine for 2 min and counterstained with 10% Mayer's hematoxylin before being dehydrated and mounted. As a negative control, the primary antibodies were replaced with normal mouse and rabbit serum.

Two independent pathologists who were blinded to the clinical status of the patients scored the stained sections under a microscope. Semi-quantitative analysis was used to score the staining results. Compared with the control, the intensity was scored as follows: 0, negative staining; 1, weak staining; 2, moderate staining; and 3, strong staining. According to the percentages of the positive-stained areas, extent of staining was scored as follows: 0, <1% positive tumor cells; 1, 1–15% 2, 15–30% 3, 30–50% and 4, >51%. The final staining scores (0–7) for FBP1 or c-Myc were the sum of the scores for percentage and the intensity. Samples with the score of ≤4 were considered to have low expression, whereas the samples having a score of >4 were considered to have high expression.^75^

### Nude mice xenograft assay and histopathology

To analyze tumor formation, the nude mice were injected with cells that had repressed FBP1. Female BALB/c nude mice were purchased from Shanghai Slac Laboratory Animal, (Shanghai, China) and maintained in microisolator cages. The animals were maintained under protocols approved by the China Care Committee Institute and this method was routinely performed as previously described.^76, 77, 78, 79^ All 12 mice were randomly assigned to two groups and underwent subcutaneous injection of 100 *μ*l of a viable cell suspension mixture (2 × 10^6^) containing 75% CNE2-NC or CNE2-siFBP1 cell suspension and 25% matrigel. When the tumors could be touched, the size of the tumor was measured by calipers daily. The tumor volume was calculated using a simplified formula: *L* × *W 2* × 0.5, where *L* is the largest dimension and *W* is the perpendicular diameter. All mice were killed on the second week after injection, and the individual tumors were weighed. All mice were killed on the second week after injection. Individual tumors were weighted and embedded in 10% paraffin. Each tissue was subjected to analyze the expression of markers (FBP1 and c-Myc) by IHC, as described previously.

### Statistical analysis

All data were analyzed using SPSS standard version 16.0 (SPSS, Chicago, USA) and GraphPad Prism version 5.0 (GraphPad Software, San Diego, CA, USA). The *χ*^2^-test or Fisher's exact test was used to assess the correlation between the clinical features and FBP1 expression. The nonparametric Spearman's rank correlation coefficient was used to evaluate the correlation between FBP1 and c-Myc expression. The Kaplan–Meier method and the log-rank test were used to determine the differences in the actual survival rates. The univariate and multivariate Cox proportional hazards models were performed to test the relative risks of FBP1 expression and other predictive variables. Data were presented as the mean±S.E.M. obtained from three independent experiments. A *P*-value of <0.05 was considered to be statistically significant.

## Figures and Tables

**Table 1:** Characteristics of the patients

**Characteristics**	**No. of patients**	**Expression of FBP1**		***P*****-value**	**Expression of c-Myc**		***P*****-value**
		**Low**	**High**		**Low**	**High**	
Patients	83	41	42		41	42	

*Age*							
Median	46						
Range	24~77						
≤46	43	24	19	0.225	9	34	0.305
>46	40	17	23		5	35	
							
*Gender*							
Female	19	2	17	0.506	2	17	0.506
Male	64	12	52		12	52	

*T classification*							
T1–T2	21	11	10	0.752	3	18	1.000
T3–T4	62	30	32		11	51	

*N classification*							
N0–N1	33	19	14	0.226	9	24	0.040
N2–N3	50	22	28		5	45	
							
*Clinical stage*							
I–II	8	4	4	<0.001	2	6	0.022
III–IV	75	37	38		12	63	

*EBV-DNA*							
<3200	44	18	44	0.931	7	37	0.804
≥3200	39	15	24		7	32	

*EBV-VCA-IgA*							
<1:160	19	7	12	0.440	4	15	0.439
≥1:160	64	30	64		19	54	

*EBV-EAIA2-IgA*							
<1 : 20	33	15	18	0.559	6	27	0.795
≥1 : 20	50	26	24		8	42	

*Locoregional failure*							
Yes	2	0	2	0.049	0	2	1.000
No	81	41	40		14	67	

*Distant metastasis*							
Yes	18	5	13	0.038	1	17	0.284
No	65	36	29		13	52	

*Tumor progression*							
Yes	19	1	18	0.172	13	51	0.172
No	64	13	51		1	18	

*Death*							
Yes	12	3	9	0.068	1	11	0.681
No	71	38	33		13	58	

Abbreviations: EBV, Epstein–Barr virus; FBP1, far upstream element-binding protein 1

**Table 2:** Univariate analysis with the COX proportional hazards for predictor of OS, and PFS of NPC patients

**Prognostic factors**	***P*-value**	**HR**	**95% CI for HR**	
			**Lower**	**Upper**
*OS*				
Age (years; >46 *versus* ≤46)	0.194	1.759	0.667	7.384
Gender (female *versus* male)	0.799	1.185	0.321	4.380
T stage (T3–4 *versus* T1–2)	0.047	2.220	0.835	8.035
N stage (N2–3 *versus* N0–1)	0.297	2.010	0.540	7.459
Clinical stage (III–IV *versus* I–II)	0.015	2.332	0.965	10.632
c-Myc (high *versus* low)	0.400	0.415	0.053	3.218
FBP1 (high *versus* low)	0.033	4.184	1.126	15.552
				
*PFS*				
Age (years; >46 *versus* ≤46)	0.365	1.019	0.978	1.036
Gender (female *versus* male)	0.048	2.654	1.008	6.989
T stage (T3–4 *versus* T1–2)	0.308	1.900	0.553	6.521
N stage (N2–3 *versus* N0–1)	0.310	1.651	0.627	4.346
Clinical stage (III–IV *versus* I–II)	0.493	2.002	0.270	15.149
c-Myc (high *versus* low)	0.141	0.220	0.029	1.654
FBP1 (high *versus* low)	0.015	3.555	1.275	9.099

Abbreviations: CI, confidence interval; FBP1, far upstream element-binding protein 1; HR, hazard ratio; OS, overall survival; PFS, progression-free survival

**Table 3:** Multivariate analysis with the COX proportional hazards for the predictor of OS and PFS of the NPC

**Prognostic factors**	***P*-value**	**HR**	**95% CI for HR**	
			**Lower**	**Upper**
*OS*				
Age (years; >46 *versus* ≤46)	0.170	0.420	0.122	1.450
Gender (female *versus* male)	0.982	1.105	0.267	3.864
T stage (T3–4 *versus* T1–2)	0.866	0.872	0.178	4.276
N stage (N2–3 *versus* N0–1)	0.401	0.553	0.139	2.220
Clinical stage (III–IV *versus* I–II)	0.048	1.900	0.984	6.399
c-Myc (high *versus* low)	0.031	0.512	0.063	4.151
FBP1 (high *versus* low)	0.039	1.405	0.680	4.268
				
*PFS*				
Age (years; >46 *versus* ≤46)	0.055	0.376	0.138	1.021
Gender (female *versus* male)	0.340	1.623	0.600	4.386
T stage (T3–4 *versus* T1–2)	0.453	0.558	0.122	2.559
N stage (N2–3 *versus* N0–1)	0.186	0.480	0.161	1.424
Clinical stage (III–IV *versus* I–II)	0.823	1.352	0.097	18.832
c-Myc (high *versus* low)	0.240	0.384	0.123	1.194
FBP1 (high *versus* low)	0.014	0.958	1.330	6.037

Abbreviations: CI, confidence interval; FBP1, far upstream element-binding protein 1; HR, hazard ratio; OS, overall survival; PFS, progression-free survival

## Abbreviations

NPC: nasopharyngeal carcinoma
FBP1: FUSE-binding protein 1
qRT-PCR: quantitative real-time PCR
siRNA: small interference RNA
MTT: 3-(4,5-dimethylthiazol 2-yl)-2,5-diphenylterazoliumbromide
DMSO: dimethyl sulfoxide
SP: side population
DDP: cisplatin;5-FU, 5-flurouracil
IHC: immuohistochemistry
OS: overall survival
PFS: progression-free survival
