# Supplementary figures and images for: Far upstream element-binding protein 1 is a prognostic biomarker and promotes nasopharyngeal carcinoma progression
Source: Cell Death Dis. 2015 Oct 15;6(10):e1920–. doi: 10.1038/cddis.2015.258 (PMC4632288; doi:10.1038/cddis.2015.258)

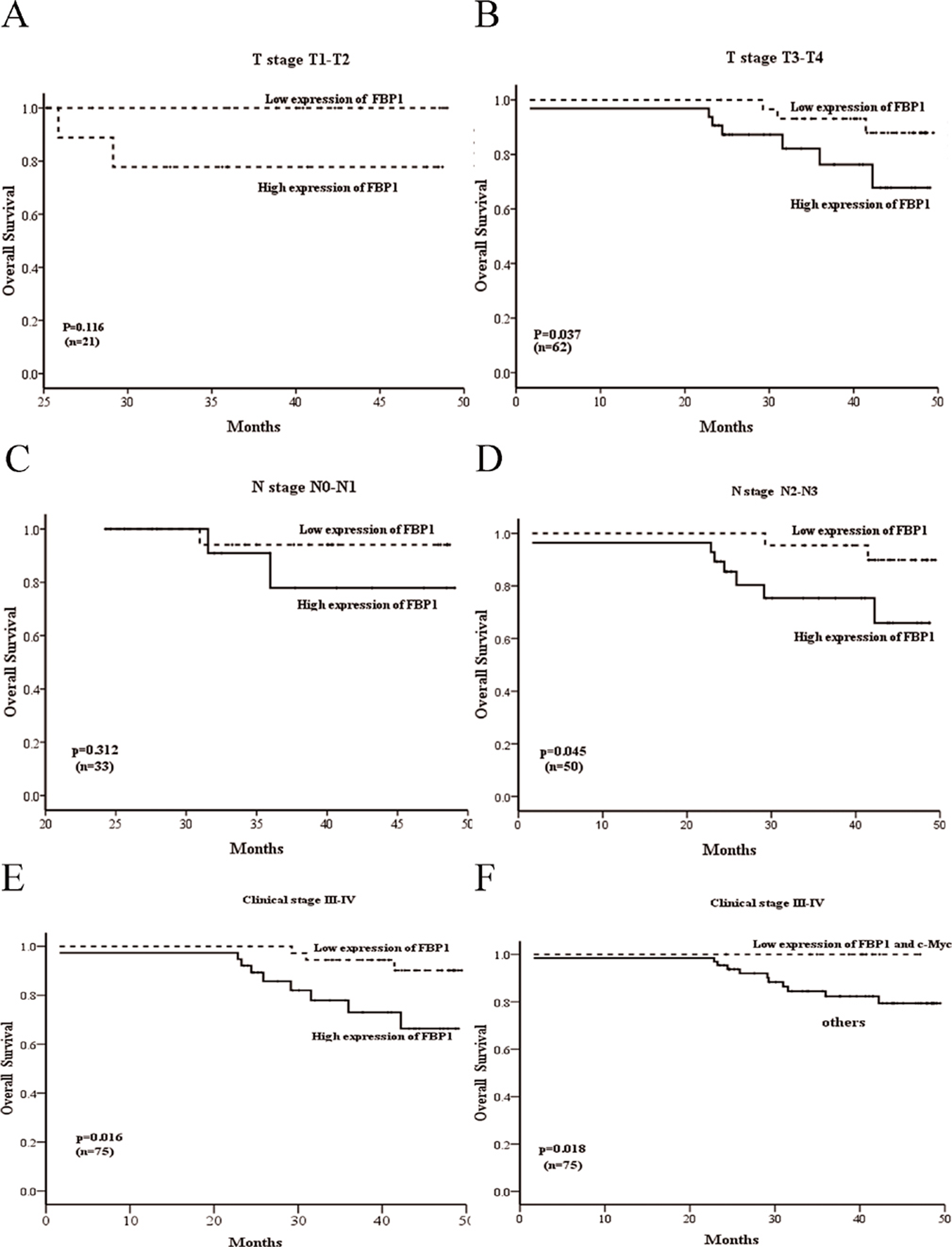

Supplement: Supplementary Figure S1 [file cddis2015258x1.tif]
